# DrugVirus.info 2.0: an integrative data portal for broad-spectrum antivirals (BSA) and BSA-containing drug combinations (BCCs)

**DOI:** 10.1093/nar/gkac348

**Published:** 2022-05-24

**Authors:** Aleksandr Ianevski, Ronja M Simonsen, Vegard Myhre, Tanel Tenson, Valentyn Oksenych, Magnar Bjørås, Denis E Kainov

**Affiliations:** Department of Clinical and Molecular Medicine (IKOM), Norwegian University of Science and Technology (NTNU), 7028 Trondheim, Norway; Department of Clinical and Molecular Medicine (IKOM), Norwegian University of Science and Technology (NTNU), 7028 Trondheim, Norway; Department of Clinical and Molecular Medicine (IKOM), Norwegian University of Science and Technology (NTNU), 7028 Trondheim, Norway; Institute of Technology, University of Tartu, 50411 Tartu, Estonia; Institute of Clinical Medicine, University of Oslo, 0318 Oslo, Norway; Department of Clinical and Molecular Medicine (IKOM), Norwegian University of Science and Technology (NTNU), 7028 Trondheim, Norway; Department of Microbiology, Oslo University Hospital and University of Oslo, 0424 Oslo, Norway; Department of Clinical and Molecular Medicine (IKOM), Norwegian University of Science and Technology (NTNU), 7028 Trondheim, Norway; Institute of Technology, University of Tartu, 50411 Tartu, Estonia; Institute for Molecular Medicine Finland, University of Helsinki, 00014 Helsinki, Finland

## Abstract

Viruses can cross species barriers and cause unpredictable outbreaks in man with substantial economic and public health burdens. Broad-spectrum antivirals, (BSAs, compounds inhibiting several human viruses), and BSA-containing drug combinations (BCCs) are deemed as immediate therapeutic options that fill the void between virus identification and vaccine development. Here, we present DrugVirus.info 2.0 (https://drugvirus.info), an integrative interactive portal for exploration and analysis of BSAs and BCCs, that greatly expands the database and functionality of DrugVirus.info 1.0 webserver. Through the data portal that now expands the spectrum of BSAs and provides information on BCCs, we developed two modules for (i) interactive analysis of users’ own antiviral drug and combination screening data and their comparison with published datasets, and (ii) exploration of the structure–activity relationship between various BSAs. The updated portal provides an essential toolbox for antiviral drug development and repurposing applications aiming to identify existing and novel treatments of emerging and re-emerging viral threats.

## Graphical Abstract

**Graphical Abstract:**
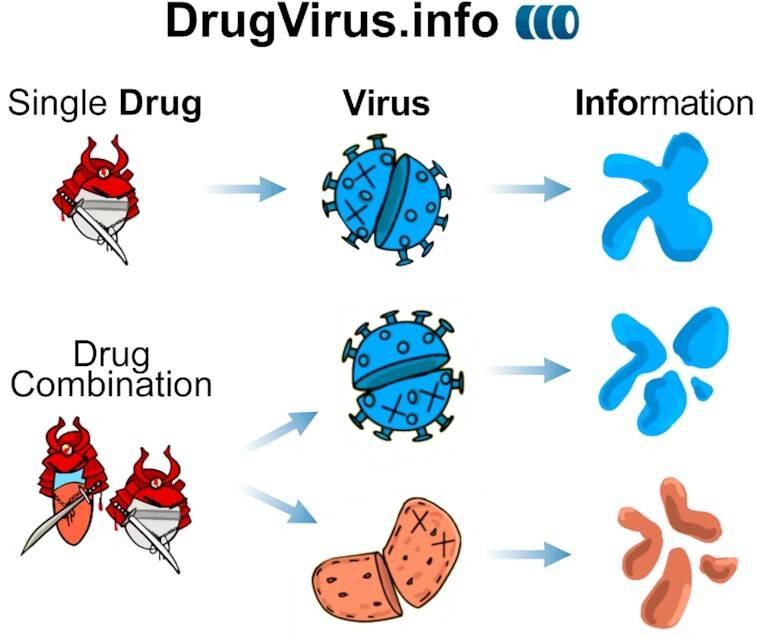
DrugVirus.info 2.0 is an integrative portal for exploration and analysis of broad-spectrum antiviral drugs and drug combinations, aiming to help in identifing existing and novel treatments of emerging and re-emerging viral threats.

## INTRODUCTION

Viral diseases consistently pose a substantial economic and public health burden. This burden is due to the ability of viruses to cross species barriers and cause unpredictable outbreaks of viral diseases in humans. The current strategy for the management of emerging and re-emerging viral diseases is heavily reliant on vaccines and antiviral treatments. While the development of novel virus-specific vaccines and drugs is often long and laborious, broad-spectrum antivirals (BSAs) and BSA-containing drug combinations (BCCs) remain timely and effective disease management options, that reduce virus transmission from human to human (1,2). BSAs inhibit replication of multiple viruses from the same or different viral families while BCCs mitigate the development of antiviral drug resistance. Moreover, synergistic BCCs contain lower concentrations of antivirals which decrease their toxicity and reduce side effects.

To date, there are hundreds of known BSAs and BCCs, and the landscape of BSA and BCC activities and virus coverage will be further interrogated and expanded (3,4). However, new solutions are urgently needed to identify the most promising hits out of thousands of potential BSAs and BCCs to prioritize their development during the critical period between the emergence of new viruses and the development of virus-specific vaccines, drugs, and therapeutic antibodies (5).

We have recently developed a DrugVirus.info 1.0 database for the exploration of safe-in-man BSAs (3). This database summarized antiviral activities and developmental status of approved, investigational and experimental 120 BSAs which inhibit 86 human viruses, belonging to 25 viral families. It was mostly used for drug repurposing/repositioning, the main approach in the search for new indications for available antivirals against emerging and re-emerging viruses, and became a popular tool among academic and clinical virologists, pharmacologists, and students. Even though the first version of the tool was a unique and extensive resource for BSAs, it was static and did not allow analysis and visualization of the user-provided antivirals. In addition, it was limited to single drugs and did not provide any information on BCCs.

Here, we present an integrative DrugVirus.info 2.0 portal for exploration and analysis of BSAs and BCCs. The updated data portal contains a comprehensive database that significantly expands the number of BSAs and incorporates BCC data. The interactive modules provide a variety of data analysis tools, including the possibility to analyze user-provided BSAs and BCCs, compare them with internal databases, and interactively explore BSAs’ structure–activity relationship. We believe that these updates should even further improve the process of discovery and development of broad-spectrum antiviral drugs and their combinations.

## MATERIALS AND METHODS

DrugVirus.info webtool allows to calculate and score BSA efficacy and toxicity as well as BCC synergy. The tool requires only the raw screening data as input (either %inhibition or %viability) and provides publication-quality visualizations and sharable report link. An example data for analysis of single BSAs and BCCs can be found on the website. A curve fitting of single-agent responses is performed using the Hill equation (i.e. the four-parameter nonlinear logistic equation):

$$\begin{equation*}g\ = \ a + \frac{{\left( {b - a} \right)}}{{\left( {1 + {{\left( {\frac{c}{d}} \right)}^n}} \right)}},\end{equation*}$$

where *g* is the response of single-agent at dose *d*, *a* is the minimum asymptote (response at zero dose), *b* is the maximum asymptote (response at infinite dose), *c* is the half-maximal effective concentration (EC_50_), and *n* is the slope (Hill coefficient) of the curve. The fitting of the dose–response curves is done using the ‘drc’ package in R (6). A ZIP synergy score for each combination is calculated using SynergyFinder, with positive and negative values denoting synergy and antagonism respectively (7,8). The compound's structure–activity dendrogram is constructed based on the structural similarity calculated by ECPF4 fingerprints (9). List of antiviral drugs was checked in the DrugBank 5.0. database (10). Illicit and exclusively veterinary drugs were not included in our database. Each of the resulting antiviral drug terms in this initial list were queried on PubMed and ClinicalTrials.org, in combination with the terms ‘virus’, ‘antiviral’ or one of the known human viruses obtained from ViralZone (11). The returned results were examined to determine if antiviral activity has been demonstrated between the drug and two or more viruses of different viral families. If antiviral activity could be established in more than two viral families, then all such drug-virus combinations would be recorded. Manual curation of >2000 PubMed articles allowed to collect SI, IC_50_/EC_50_ and CC_50_/TC_50_ values for BSAs and synergy scores (synergistic, additive or antagonistic) for BCCs. The corresponding articles PubMed ids are available in the database. D3 v6 JavaScript library was utilized for the implementation of interactive visualizations, a front end is written with PHP and HTML while the backend is implemented with R. The feedback form is also available on the website and we ask users to continue leaving their suggestions for future improvements, to make DrugVirus.info even more comprehensive, interactive and user-friendly.

## RESULTS

**Figure 1.**
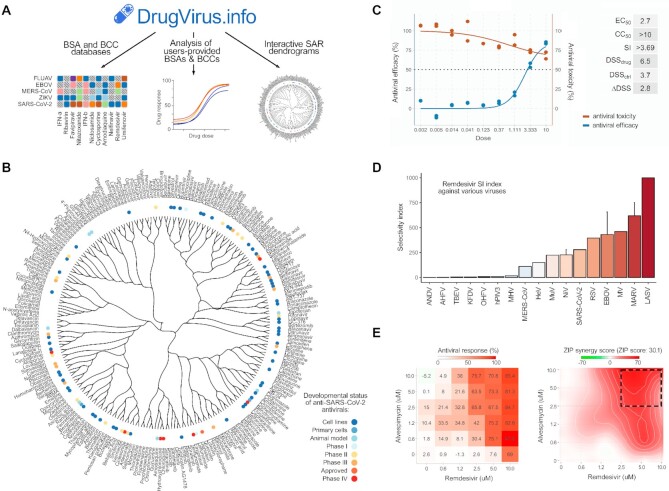
Overview of DrugVirus.info 2.0 portal. (**A**) The portal comprises three major components BSA and BCC databases, module for analysis and comparison of user-provided BSAs and BCCs data, and structure–activity relation (SAR) module. (**B**) The circular dendrogram shows the SAR of 250 BSAs and stages of their development against SARS-CoV-2. (**C**, **D**) Example of analysis of user-provided data for single BSA and comparison of resulting selectivity indexes (SI = CC_50_/EC_50_) obtained for other viruses. (**E**) Example of automated calculation and visualization of BCC synergy score analysis.

The DrugVirus.info 2.0 portal includes three major components (i) BSA and BCC interactive exploration databases, (ii) structure–activity relationship (SAR) dendrogram tree of BSAs, as well as (iii) user-provided BSA and BCC data analysis modules (Figure 1A).

### BSA and BCC interactive exploration databases

In a second release of DrugVirus.info, we extended an available information on BSAs (from 116 to 255 compounds), while still focusing on those antivirals, which have been already tested in human as antivirals, antibacterials, antiprotozoals, anthelmintics, etc. In addition, we created a second database providing information on BSA-containing drug combinations and included 400 combinations. The drug annotations were obtained from PubChem, DrugBank, DrugCentral, PubMed and clinicaltrials.gov databases (10,12,13). The information on virus families was exported from Virus Pathogen Database and Analysis Resource (14). The database summarizes activities and developmental status of BSAs. We also added information for EC_50_/IC_50_, CC_50_/TC_50_ and selectivity index (SI values) for all BSAs where available. Similarly, we added synergy results (synergistic, additive, or antagonistic) for BCCs. Both databases allow interactive exploration of virus-BSA interactions and include information on BSA targets. The databases provide a comprehensive and unique resources for exploration of BSAs and BCCs.

### Structure-activity relationship dendrogram of BSAs

DrugVirus.info 2.0 implements an interactive exploration module that visualizes structure–activity relationship (SAR) based on DrugVirus.info BSA database (Figure 1A). SAR dendrogram displays the similarity between chemical structures of BSAs as a hierarchically-clustered tree, where each compound forms the node in the tree annotated with the respective developmental status of the compound against the user-selected virus (bubbles around the tree). As an alternative to tree dendrogram, one may also visualize a cluster dendrogram and select other viruses for dendrogram annotation. The compound structures for each BSA were obtained in the form of SMILES from PubChem database (12). For example, Figure 1B shows the circular dendrogram representing SAR of 250 BSAs and stages of their development against SARS-CoV-2. Sub-clusters of active BSAs indicate the space for structurally similar BSAs which could be developed further for treatment of SARS-CoV-2 infection. Users may also upload their own chemical structures and the portal will automatically recreate an interactive compound similarity clustering dendrogram. Thus, users can investigate the structural relationships between the compounds of interest and available BSAs and interactively compare their developmental phases, reducing the time required for manual analysis in antiviral drug development and repurposing applications.

### BSA and BCC data analysis modules

DrugVirus.info 2.0 allows to calculate and score BSA efficacy and toxicity. For example, Figure 1C shows selectivity indexes (SI = CC_50_/EC_50_) obtained for remdesivir against SARS-CoV-2 and other viruses. It suggests that remdesivir is active against many RNA viruses. The tool requires the raw screening data as input (%inhibition or %viability) and fits the dose–response curves using the Hill equation. In case, the replicates are provided, all of them will be utilized for fitting resulting in more robust curve fits. Next, DrugVirus.info summarizes dose–response relationship into single metric including EC_50_, CC_50_ (in case the toxicity data is provided), selectivity index (SI = CC_50_/EC_50_) and drug sensitivity score (DSS), that represents a normalized version of standard AUC (15,16). In addition, the module allows interactive comparison of resulting data (Figure 1D). DrugVirus.info 2.0 also calculates and scores BCC synergy. For this, it also utilizes the raw screening data as input and calculates a ZIP synergy score for each combination using SynergyFinder, with positive and negative values denoting synergy and antagonism respectively (7,8). As an output, the tool provides publication-quality visualizations and sharable report link (Figure 1E).

## CONCLUSIONS

We developed a DrugVirus.info 2.0, an integrative exploration and analysis portal of BSAs and BCCs. The latest update of the portal provides information on 255 BSAs and 407 BCCs and allows their interactive exploration and comparison. Designed to be accessible for researchers without any programming skills, DrugVirus.info 2.0 implements user-friendly interactive analysis and visualization modules that automatically analyses user-provided antiviral single and combination drug response data. The data generated and/or analyzed using DrugVirus.info 2.0 may help to unveil new insights into the virus-host interactions and the underlying principles that determine pan- and cross-family activities of BSAs and BCCs, decipher their mechanisms of action, and understand what makes some drugs act synergistically, something that is still largely unknown in the medical science community.

## DATA AVAILABILITY

DrugVirus.info 2.0 web portal is freely accessible to users without any login requirements at https://drugvirus.info. Extensive documentation that explains all the features is available at https://drugvirus.info/tech_doc.
